# Perspectives of patients on the role of general practice pharmacists: a systematic review and meta-synthesis of qualitative studies

**DOI:** 10.1186/s12875-025-02787-0

**Published:** 2025-03-31

**Authors:** Abrar H. F. Hassan, Heather E. Barry, Carmel M. Hughes

**Affiliations:** 1 Primary Care Research Group, School of Pharmacy, Medical Biology Centre, Queen’s University Belfast, 97 Lisburn Road, Belfast, BT9 7BL Northern Ireland, UK; 2 Department of Clinical Pharmacy, College of Pharmacy, Jazan University, Jazan, Saudi Arabia

**Keywords:** Primary care, General practice pharmacists, Patients, Qualitative synthesis

## Abstract

**Background:**

There is a scarcity of research about patients’ perspectives on the role of general practice pharmacists (GPPs). In this review, we aimed to compile qualitative evidence of patients’ perspectives regarding the role of GPPs.

**Methods:**

A systematic, qualitative meta-synthesis was undertaken. A comprehensive search was conducted on six databases. Additionally, the reference lists of included studies were searched. Findings and verbatim quotes were extracted from the included studies and were analysed using thematic synthesis. The Critical Appraisal Skills Programme (CASP) checklist was employed to evaluate the quality of the included studies. The GRADE-CERQual approach was utilised to evaluate confidence in the findings.

**Results:**

Ten qualitative studies were included. Four main themes were identified: awareness of the GPP (patients were unaware of the GPP’s role), accessibility to the GPP (some patients had difficulties arranging appointments with GPPs), benefits and challenges (medication review conducted by GPPs reaffirmed patients’ trust in taking their medicines, although some were dissatisfied with the medication review process), and GPP integration into general practice (successful integration of GPPs was attributed to their skills and teamwork). The included studies satisfied all or at least seven out of the ten criteria of the CASP checklist. GRADE-CERQual indicated high confidence for one theme, and moderate confidence for three themes.

**Conclusions:**

This systematic review and meta-synthesis of qualitative studies provides valuable insights into patients’ perspectives on the role of GPPs. The findings highlight both positive aspects and challenges associated with GPP integration into primary care, including concerns about role awareness and accessibility. These findings suggest that while GPPs can add value to general practice teams, there is a need for improved patient education about the GPP role and enhanced accessibility to maximise the potential benefits of the GPPs.

**Clinical trial number:**

Not applicable.

**Supplementary Information:**

The online version contains supplementary material available at 10.1186/s12875-025-02787-0.

## Background

Research has highlighted pressures within general practice in the United Kingdom (UK) and the growing demands for patient care for those with multimorbidity and polypharmacy [1–5]. To overcome these pressures, the UK General Practice Forward View (GPFV), published in 2016, offered a new integrated model of general practice in the UK [6]. This model comprised five specific and practical steps: (1) fund primary care by over £12 billion annually by 2020/21; (2) support for GPs and primary care teams via integration of healthcare and administrative staff such as mental health therapists, clinical pharmacists, nurses and receptionists; (3) lessen practice workload pressures through introduction of a novel practice resilience programme; (4) use technology such as approval of specific applications (apps) for clinicians and patients; (5) establish an improvement programme by redesigning care, encouraging patient self-care, utilising workforce skills and enabling healthcare professionals (HCPs) to work in different practices [6]. One suggestion from the GPFV was the integration of pharmacists in general practice (also called general practice pharmacists; GPPs) [6]. This integration across the UK has been funded and piloted by different plans since 2015, and research has been undertaken to evaluate this practice development [3, 7, 8]. GPPs are qualified experts in medicines and have diverse knowledge and skills which has led to improved access to healthcare and reduced appointment wait times in general practice [1].

Studies have shown that stakeholders hold positive views about the role of GPPs [1, 9–15]. For example, the integration of GPPs into general practice has led to a reduction in GP workload, improved patient safety, and generated cost savings on medicines [1, 9–15]. The most common activities undertaken by GPPs in general practice were medication reconciliation and medication reviews [1, 14]. Currently, there is limited evidence available on the views of patients about the GPP’s role [1, 14, 16]. Previous studies that investigated patients’ views have recommended further exploration of this area as there are some knowledge gaps on patients’ views of the GPPs which have not been addressed such as uncertainty regarding the role of GPPs and if contact with GPPs would be continuous [11, 15, 17, 18].

Therefore, the aim of this study was to address these gaps by synthesising qualitative research findings on patients’ perspectives of the GPP’s role. The objectives were to:

- Investigate patient awareness of the GPP’s role in general practice.
- Investigate patients’ perspectives on access to and communication with GPPs.
- Identify barriers and enablers for patients consulting with GPPs.
- Identify patient information needs on the role of GPPs in general practice.

## Methods

### Methodology

In this study, a qualitative meta-synthesis (qualitative synthesis) method was used to systematically assess and combine the findings of qualitative research that investigated patients’ perspectives on the GPP’s role [19]. Stern and Harris (1985) coined the phrase “qualitative meta-analysis” to describe a meta-synthesis of qualitative findings in nursing literature [20].

### Question formulation

Several frameworks have been established to aid the formulation of a research question for qualitative synthesis [21]. Depending on the elements (keywords) of the question that need to be answered by qualitative synthesis, different frameworks can be utilised [21]. The following two frameworks have often been used in qualitative synthesis [22, 23]:

- SPICE (Setting, Perspective, Intervention or Phenomenon of Interest, Comparison, Evaluation) [22].
- SPIDER (Sample, Phenomenon of Interest, Design, Evaluation, Research type) [23].

The SPIDER framework was used in this study because the SPIDER terminology was more compatible with the keywords of the research question (what are patients’ views on the GPP role in general practice? ) as shown below:

Sample: Patients who have experience of the GPP role or patients who have experience of the activities/services delivered by the GPP or have been in contact with the GPP.

Phenomenon of Interest: Patients’ views of the GPP role in general practice.

Design: Specified types of qualitative data collection (e.g. focus groups, semi-structured/structured interviews) and analysis (e.g. thematic analysis).

Evaluation: Patients’ views, experiences, opinions, thoughts, ideas, perceptions, or perspectives of the GPP role qualitatively described.

Research type: Primary qualitative studies and mixed-method studies with a qualitative component.

### Search strategy

Six electronic databases were searched during July 2022-June 2024 including Medline, Embase, Scopus, Web of Science, International Pharmaceutical Abstracts (IPA), and Cumulative Index to Nursing and Allied Health Literature (CINAHL) from date of inception of each database to June 2024 to find relevant studies on patients’ perspectives of the role of GPPs. The search strategy [see Additional file 1] was developed with assistance of a subject librarian from Queen’s University Belfast Library and used to conduct the searches.

### Inclusion and exclusion criteria

Studies had to address views of patients about the GPP role in general practice. Studies had to report primary, empirical, peer-reviewed research, published in the English language, and have used qualitative methods for both data collection (e.g. focus groups, interviews) and analysis (e.g. thematic analysis). Theses and grey literature were not included in this review; additionally, in order to include the richest qualitative findings [data that give more detailed explanation of the relevant phenomenon], survey studies with open (free text) comment sections were excluded [21]. When studies used mixed method approaches, the qualitative data only were extracted from the study. When it was not possible to differentiate between quantitative and qualitative components of analysis, the study was excluded. Moreover, reference lists of included studies in this review were searched to identify other potentially eligible studies.

### Study selection

The studies incorporated in this review were imported into EndNote 20 (Clarivate, 2013), and duplicates were eliminated. CMH and AHFH conducted title screening of all selected studies to eliminate studies irrelevant to the research question. Likewise, both CMH and AHFH conducted screening of the titles and abstracts of all remaining studies to ascertain their compliance with the inclusion criteria. Subsequently, one author (AHFH) conducted a review of the full texts of studies following titles and abstract screening to ascertain their eligibility for inclusion in the final analysis. The Preferred Reporting Items for Systematic reviews and Meta-Analyses (PRISMA) flow diagram was used to report the systematic search process [24].

### Data extraction

The data extraction process involved a two-step process: extracting contextual information (such as research aim, participants, study setting, method of data collection, and analysis) and extracting qualitative findings [25]. This review referred to data extraction forms from previous studies as well as literature related to extracting qualitative evidence which informed the development of the form of this review [26–28]. The data collection form was not piloted before use.

### Quality appraisal of included studies

The Critical Appraisal Skills Programme (CASP) Checklist for qualitative studies was used to assess the quality of the included studies and the methodology used [21, 29, 30]. The CASP Checklist is the most utilised tool to appraise quality of the qualitative studies [21, 30].

### Data analysis and synthesis

This study used the thematic synthesis approach of Thomas and Harden to analyse data extracted from primary qualitative studies [21, 31]. The process involved three key steps: line-by-line coding, developing descriptive themes, and generating analytical themes [31]. The first step entailed labelling findings and creating a list of descriptive codes [31–33]. In the second step, codes were grouped into descriptive themes and subthemes based on similarities and differences of the codes [31]. The third step involved interpreting descriptive themes into keywords related to the question of this study [31]. The research team discussed the synthesis of findings and examined the derived themes to reach final agreement.

### Assessment of confidence in findings

The GRADE-CERQual approach was applied to assess confidence of findings generated from this study [21, 34, 35]. This approach involves listing individual review findings and assessing confidence based on four components: methodological limitations, coherence, adequacy of data, and relevance, and making a judgement about the presence or seriousness of concerns regarding the four components in each finding [34, 35].

### Reporting and dissemination of results

The ENTREQ (Enhancing transparency in reporting the synthesis of qualitative research) checklist was used to report this study, which can help researchers identify stages commonly associated with qualitative research synthesis [see Additional file 2] [21, 36]. This study was registered with the International prospective register of systematic reviews (PROSPERO; registration number CRD42023423623) after finalising the study protocol and screening studies for inclusion [37]. Ethical approval was not required as this study was a secondary analysis of published data.

## Results

From July 2022 to August 2022, 29 records were identified through database searches. No additional records were found between August 2022 and May 2023. In a final search conducted from May 2023 to June 2024, two further records were retrieved from databases, resulting in a total of 31 records identified through database searches. After screening titles and abstracts, two records were excluded while 29 were assessed for eligibility using full text. Twenty-one records were excluded, and eight records were retained.

**Fig. 1:**
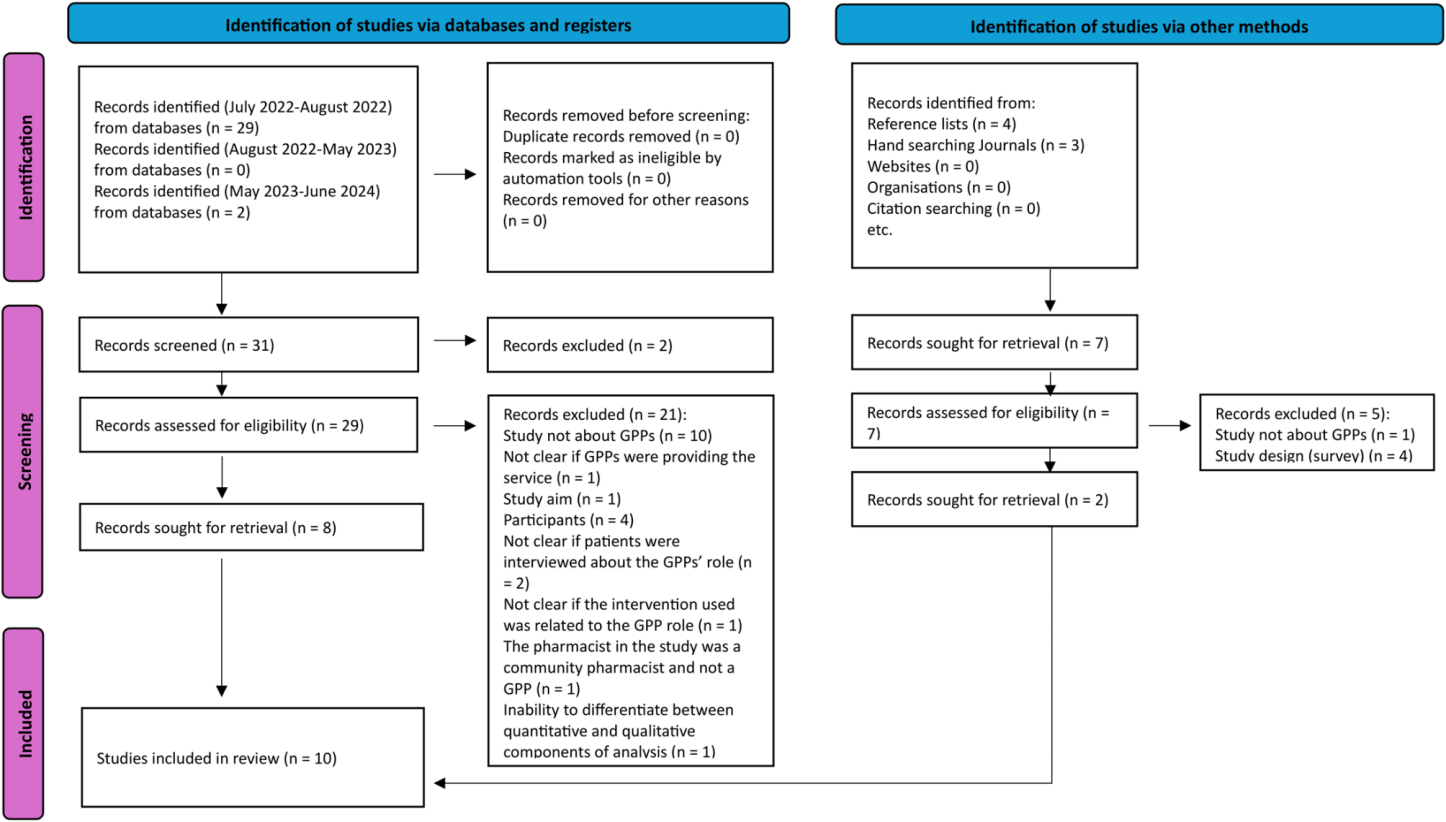
PRISMA 2020 flow diagram for new systematic reviews which included searches of databases, registers and other sources

Additionally, four records were identified from reference lists, and three from hand-searching journals. No records were found from websites, organisations, or citation searching. This brought the total number of records identified through other methods to seven which were retained following the screening of titles and abstracts. These seven records were assessed for eligibility using full text and five were excluded. Thus, the total number of retrieved records via other methods was two. Therefore, the overall number of records identified from both databases and other methods was 10, representing 10 studies included in this review. The PRISMA 2020 flow diagram [Fig. 1] illustrates the selection and exclusion of studies. Table 1 summarises the features of the ten included studies, including the country of the study, purpose, number of participants, setting, method of sampling, data collection, and data analysis approach.

**Table 1:** Characteristics of included studies

Author and year	Country where study took place	Aim	Number of patient participants	Setting	Method of sampling	Method of data collection	Data analysis approach
Ryan et al. [11]	UK	To explore the experiences of stakeholders of pharmacy services in general practices provided by GPPs	9	Eight general practices in the Ealing GP Federation, West London	Purposive sampling	Semi-structured interviews	Thematic analysis
Karampatakis et al. [17]	UK	To conduct an extensive investigation of patients’ perceptions of pharmacists in general practice	20	Three general practices in West London, Surrey and Berkshire	Purposive sampling	Semi-structured interviews	Thematic analysis
Mann et al. [18]	UK	To examine the patient view of pharmacists in patient-facing roles in general practice in the UK	34	Selected general practices across the UK	Purposive and opportunistic sampling	Focus group interviews	Thematic analysis
Ahn et al. [38]	Australia	To investigate patients’ views on understanding home medicines review (HMR) as well as notable benefits and difficulties of HMR which was delivered by a pharmacist in the general practice	15	One large general practice	Purposive sampling	Semi-structured interviews	Thematic analysis
Deeks et al. [39]	Australia	To explore stakeholder (patients, practice staff and community pharmacists) perspectives on the advantages, difficulties, and facilitators of incorporating pharmacists into general practice	7	Three general practices in the Australian Capital Territory (ACT)	Purposive sampling	Semi-structured interviews	Thematic analysis
Donaghy et al. [40]	UK	To explore patients’ views on the changes in general practice in Scotland since the inception of the new contract [the Scottish GP contract, implemented in April 2018, intended to improve quality of care by expanding the multidisciplinary team (MDT) and allowing GPs to focus on patients with complex needs]	30	Twelve general practices in Scotland	Purposive sampling	Semi-structured interviews	Thematic
McCahon et al. [41]	UK	To explore patient experiences of medication review including the processes and activities that led up to and shaped the review	21	Ten general practices in Bristol	Purposive sampling	In-depth interviews	Thematic
Petty et al. [42]	UK	To obtain patients’ views of a pharmacist-conducted medication review clinic, run in their general practice	18	General practices in Leeds Health Authority area	Purposive sampling	Focus groups	Thematic
Stewart et al. [43]	UK	To investigate the perspectives of pharmacist supplementary prescribers, their linked independent prescribers (doctors) and patients, across a range of settings in Scotland on pharmacist prescribing	18	Primary and secondary care settings in six NHS Health Board areas in Scotland	Purposive sampling	Telephone interviews	Framework
Tan et al. [44]	Australia	To examine general practice staff, pharmacist and patient experiences with pharmacist services in Australian general practice clinics within the Pharmacists in Practice Study (PIPS)	18	Two general practices in Melbourne, Australia	Purposive sampling	Semi-structured interviews	Thematic

The ten included studies were published between 2003 and 2024 and were conducted in two countries: the UK and Australia. The total number of patient participants was 190, with sample sizes across studies ranging from 7 to 34. Most studies used semi-structured interviews for data collection, and thematic analysis was the most common analytical approach.

This review identified four major themes: awareness of the GPP, accessibility to the GPP, benefits and challenges for patients, and GPP integration into general practice (see Table 2).

**Table 2:** Representative extracts of the review themes

Theme	Extract	Reference
Awareness of the GPPs	Most patients exhibited a lack of awareness regarding the integration of pharmacists within the general practice. As reported by a patient, ‘*I had been coming to this practice for 24 years and I didn’t know that there was a pharmacist here. It’s possibly not my fault*,* they don’t advertise*,* promote*,* they don’t explain enough… I got a text [message] saying make an appointment with the pharmacist… [I was thinking] What are they talking about? Where?*’ Patients were not sure about the purpose of seeing the GPP and were reluctant to attend the appointment with the GPP. As indicated by one patient, ‘*The doctor just told me ‘I’m going to do an appointment for you to see the pharmacist’ and that’s it and I haven’t any idea what’s going on*,* just they told me ‘bring all your tablets with you*.’	Karampatakis et al. [17]; Donaghy et al. [40]; McCahon et al. [41]; Tan et al. [44]
Accessibility to the GPPs	Patients reported that it was easier to get an appointment with a GPP within a week compared to a GP. ‘*I got this appointment quite quick*,* so*,* whereas if it was a doctor I think it was another 2 weeks or something*,* which*,* because it was only to review my medication*,* I felt that’s quite a long time to wait just for that*,* so this is a good way of doing it*.’ The fact that GPPs were only accessible for a specific number of hours each week and on specific days was considered a drawback of GPPs’ services in the practice by some patients, *‘…but she might not be there on the day that you need them*.’	Ryan et al. [11]; Tan et al. [44]
Benefits and challenges for patients	An advantage highlighted by some patients was the opportunity for their medicines to be examined holistically during the HMR, ‘*It’s a very good plan in the sense that the patient gets to have a real comprehensive overview of all the medicines they’ve been prescribed…it’s always good to refer to somebody who is more trained*,* particularly in the various effects of medicines*.’ Some patients felt it was difficult to attend their appointments with the GPP, ‘…*just another one of the millions of other appointments I have regarding what’s going on with me at the moment*.’	Ahn et al. [38]; Tan et al. [44]
GPP integration into general practice	Patients thought that the GPP’s personality and skills had an impact on how successfully they integrated into the general practice. ‘[The practice pharmacist] *was very*,* very patient and she gave the impression she really knew what she was talking about…she could explain everything*.’ Patients expressed satisfaction with a combination of the GPP and GP providing care. ‘*So* [pharmacist CP1] *has been working with* [nurse 1] *and* [healthcare assistant 1] *first of all*,* in like a threesome*,* to get my tachycardia so that it wouldn’t be a problem. So*,* I have been seeing her [pharmacist CP1] regularly. I find it is a combination of everybody really because I can’t remember the last time I saw the doctor. It was either* [pharmacist CP1], *or* [nurse 1]. *Between the three of you*,* you have all sorted me. It’s very rare I bother the doctor. You are doing him out of a job*.’	Mann et al. [18]; Tan et al. [44]

NHS: The National Health Service; HMR: Home Medicine Review

### Awareness of the GPP

Patients were found to be unaware of the GPP’s role and the difference between the role of the GPP and the community pharmacist [11, 17, 18, 38–44]. Patients reported this lack of awareness had led to misunderstandings and hesitancy to book or attend appointments with GPPs [11, 18, 44]. To increase awareness, various promotion strategies were suggested, including television advertisements, messages on practice websites, social media accounts, waiting room screens, and creating visible consultation spaces for GPPs [17].

### Accessibility to the GPP

Accessibility to GPPs varied across the included studies. Some patients reported shorter waiting times than those for GPs, while others reported issues arranging appointments with GPPs who provided services at multiple practices [11, 17, 18]. Patients considered GPPs’ limited availability in general practice and occasional cancellation of appointments with patients as disadvantages [43].

### Benefits and challenges for patients

Patients were generally satisfied with the GPP’s approach and the information they received during consultations [18, 39–41]. However, patients reported insufficient time commitment and poor attitude from GPPs [38, 39]. For instance, some patients reported that they did not discuss their medicines during the medication review with the GPP and received dismissive answers to their enquiries [38]. Nonetheless, some patients reported better health literacy (defined as the ability to access, comprehend, assess, and use health information and services to make informed decisions about health and well-being) after a medication review with a GPP [38, 45]. Additionally, patients indicated that medication review served to reaffirm their confidence in taking their medicines by providing detailed information about patients’ medicines [39].

Some patients expressed dissatisfaction with the medication review process with unrealistic expectations such as expecting the GPP to discontinue their medications or cure their illness [18, 41, 42]. These patients reported their concerns to their GPs, who also expressed disappointment with the medication review process [38, 39, 42]. Some patients experienced a six-month delay in receiving a medication review report, which their GPs deemed ‘useless’ [39]. Despite these challenges, patients believed GPPs increased their knowledge and awareness of their medicines, offered reassurance, promoted medication adherence, rationalised drug therapy, and improved health outcomes [43].

### GPP integration into general practice

Patients reported that GPPs’ integration into general practice was attributed to their skills and teamwork [43]. Patients believed that GPPs needed good interpersonal and communication skills [43]. The absence of judgemental attitudes of GPPs created a sense of comfort and ease [18, 19, 43]. Patients felt secure, comforted, and at ease knowing their issues had been handled [18, 19, 43]. Patients also described their relationship with GPPs and GPPs’ relationship with their GPs and other practice staff as largely positive [43]. Patients appreciated the opportunity to speak with GPPs about their prescribed medicines and felt that consulting with the GPPs would not negatively impact their relationship with their GPs [43].

The study quality assessment revealed that most studies met the CASP checklist’s criteria, but three did not provide sufficient information about participant recruitment [11, 18, 43], five did not discuss potential bias during data collection, and one did not provide an in-depth description of the data analysis process [11, 39, 41, 42, 44]. The GRADE-CERQual approach assessed review themes for confidence, with one theme rated high and three as moderate. The overall assessments are summarised in a summary of qualitative findings in Table 3.

**Table 3:** Confidence in the evidence from reviews of qualitative research (CERQual)

Review themes	Studies contributing to the review themes	Assessment of methodological limitations	Assessment of relevance	Assessment of coherence	Assessment of adequacy	CERQual assessment of confidence in evidence	Explanation of CERQual assessment
**Awareness of the GPP**	Ryan et al. [11]; Karampatakis et al. [17]; Mann et al. [18]; Ahn et al. [38]; Deeks et al. [39]; Donaghy et al. [40]; McCahon et al. [41]; Petty et al. [42]; Stewart et al. [43]; Tan et al. [44]	Minor methodological limitations. Three studies had minor methodological limitations (Stewart et al. [43]; Ahn et al. [38]; Mann et al. [18])	No limitations identified	No limitations identified	No limitations identified	High	This finding was graded as high confidence because of minor concerns regarding methodological limitations
**Accessibility to the GPP**	Ryan et al. [11]; Karampatakis et al. [17]; Mann et al. [18]; Tan et al. [44]	No limitations identified	No limitations identified	Moderate concerns about coherence (data inconsistent across all studies as only four studies reported this finding)	Moderate concerns about adequacy (only four studies reported this finding)	Moderate	This finding was graded as moderate confidence because of moderate concerns on coherence and adequacy of data

Review themes	Studies contributing to the review finding	Assessment of methodological limitations	Assessment of relevance	Assessment of coherence	Assessment of adequacy	CERQual assessment of confidence in evidence	Explanation of CERQual assessment
**Benefits and challenges for patients**	Ahn et al. [11]; Karampatakis et al. [17]; McCahon et al. [41]; Petty et al. [42]; Stewart et al. [43]; Tan et al. [44]	No limitations identified	No limitations identified	Moderate concerns about coherence (data inconsistent across all studies as only five studies reported this finding)	Moderate concerns about adequacy (only five studies reported this finding)	Moderate	This finding was graded as moderate confidence because of moderate concerns on coherence and adequacy of data
**GPP integration into general practice**	Ryan et al. [11]; Karampatakis et al. [17]; Mann et al. [18]; Deeks et al. [38]; Tan et al. [44]	No limitations identified	No limitations identified	Moderate concerns about coherence (data inconsistent across all studies as only five studies reported this finding)	Moderate concerns about adequacy (only five studies reported this finding)	Moderate	This finding was graded as moderate confidence because of moderate concerns on coherence and adequacy of data

## Discussion

Qualitative meta-synthesis was conducted to combine qualitative findings on patients’ views of the GPP role to identify themes, theories, or concepts related to this area [46, 47]. The findings revealed that patients lacked knowledge about GPPs’ existence and activities [38–44]. In an interview study in the UK, general practice staff (GPs, pharmacists, practice managers, practice nurses and receptionists) showed enhanced understanding of GPPs’ responsibilities, but some staff and patients still had limited awareness of GPPs and their role [11]. Moreover, the results of a study that compared the establishment of three non-medical roles in general practice– practice pharmacists, physician associates, and advanced practitioners– indicated that adding new roles can have both intended and unintended effects [48]. Specifically, ambiguity about the purpose of new roles in general practice, as well as the difficulties around role definition and setting professional boundaries, influenced the degree to which these roles were incorporated in general practice settings [48]. Therefore, promotion of the GPP’s presence to patients, general practice staff and the public may improve the limited awareness that patients have about GPPs [11].

Patients’ experiences with arranging appointments with GPPs varied, with some finding it easier than arranging appointments with GPs [11, 17, 18]. However, patients who had negative experiences were disappointed with the lack of availability of GPPs working at multiple practices and the inability to contact GPPs directly [11, 17, 18]. The availability of GPPs to have consultations with patients was also a concern, with the role evolving over time and GPPs taking on additional responsibilities such as independent prescribing and running review clinics for patients with long-term conditions [49]. This current review suggested that availability of full-time GPPs in general practices and direct communication with GPPs were important to enhance accessibility [1].

Patients reported benefits and challenges when consulting with GPPs. Benefits included identifying medicines-related problems, improving patient safety, knowledge and understanding about medicines [8, 38, 43]. However, challenges were related to medication reviews (e.g. short-duration medication reviews) and GPPs’ skills (e.g. GPPs’ failing to document patient-related information) [17, 42]. Pharmacists need specific skills and training to be better integrated into primary care teams, such as teamwork, patient evaluation, care planning, documentation, and evidence-based decision-making [50]. Pharmacists should assess their specific learning needs and consider participating in professional development programmes such as the patient care skills development programme, which is offered by the Canadian Pharmacists Association [50]. Furthermore, a recent study has developed a core set of clinical skills needed for prescribing pharmacists to work in general practice such as measuring heart rate (radial pulse) and assessing respiratory rate [50]. This highlights the necessity of GPPs having access to specific training to improve the provision of their services in general practice [51].

Good interpersonal and communication skills of GPPs are required for better integration in practice [44]. A research paper has shown that a GPP’s personality may impact on their integration and the role they provide in the practice [13]. For GPPs to have the greatest influence, they must determine where they are most needed within the practice multidisciplinary team and incorporate themselves within it, particularly by identifying the medication-related needs of this team and the patient population to facilitate the provision of GPP services [13, 50]. Patients expressed satisfaction with the GPP’s relationship with them, with GPs, and with other practice staff [44]. A study in New Zealand showed that GPs wanted to collaborate with pharmacists in general practice, and that pharmacists and GPs should work together through improved information exchange and increased communication [52]. The presence of pharmacists in general practice enhanced communication and relationships between community pharmacies and general practices, and practices that employed pharmacists were more likely to consider communication from community pharmacies to investigate medicines issues such as overuse of hypnotics [12, 52].

### Strengths and limitations

The method utilised in this review, qualitative meta-synthesis, is regarded as an important strategy for influencing healthcare and pharmacy research, practice, and policy [19, 47]. Qualitative meta-synthesis assists HCPs and policymakers in better understanding patients’ lived experiences, allowing them to make more informed decisions [19, 47] Six databases were thoroughly searched for relevant material. The CASP checklist was used to assess the quality of every study included in this review. Many of the included studies had excellent methodological quality overall. In addition, the GRADE-CERQual technique was utilised to determine confidence in the review findings.

However, the only studies found through our searches were written in English. Thus, it is possible that relevant research has been published in other languages but has yet to be found. Furthermore, only ten papers were included in this review, demonstrating the lack of qualitative studies on patients’ perspectives of the GPP’s role. Furthermore, the absence of studies from countries other than the UK and Australia limited the relevance of our findings to settings with diverse economic backgrounds and healthcare systems.

## Conclusions

This review explored patients’ perspectives of the GPP’s role, focusing on awareness, accessibility, benefits, challenges, and integration. Findings suggested patients were largely unaware of the existence of GPPs and further research is needed to understand the GPP role and its impact on patient outcomes.

## Electronic supplementary material

Below is the link to the electronic supplementary material.

**Additional file 1**: The search strategy.

**Additional file 2**: The ENTREQ checklist.

## Data Availability

The datasets used during the current study are available from the corresponding author on reasonable request.

## Abbreviations

APP: Application
CASP: Critical Appraisal Skills Programme
CINAHL: Cumulative Index to Nursing and Allied Health Literature
ENTREQ: Enhancing transparency in reporting the synthesis of qualitative research
PROSPERO: International prospective register of systematic reviews
GP: General practitioner
GPFV: General Practice Forward View
GPP: General practice pharmacist
HCP: Healthcare professional
IPA: International Pharmaceutical Abstracts
PRISMA: The Preferred Reporting Items for Systematic reviews and Meta-Analyses
SPICE: Setting, Perspective, Intervention or Phenomenon of Interest, Comparison, Evaluation
SPIDER: Sample, Phenomenon of Interest, Design, Evaluation, Research type
UK: United Kingdom
