# Supplementary material for: Perspectives of patients on the role of general practice pharmacists: a systematic review and meta-synthesis of qualitative studies
Source: BMC Prim Care. 2025 Mar 31;26:90. doi: 10.1186/s12875-025-02787-0 (PMC11956316; doi:10.1186/s12875-025-02787-0)
Supplement: Supplementary file 1 — Additional file 1: The search strategy. [file 12875_2025_2787_MOESM1_ESM.docx]

**Search strategy for databases searched in this study**

| **Database** | **Search strategy and keywords** |
| --- | --- |
| **Medline** | (patient* and (experience* or view* or opinion* or thought* or idea* or perception* or perspective*) and (pharmacy or pharmacist* or pharmac* or clinical pharmacist* or pharmacy service* or clinical pharmacy service* or primary healthcare pharmacist* or primary care pharmac* or practice-based pharmacist* or general practice pharmac* or practice pharmacy professional or non?dispensing pharmacist*) and (implement* or integrat* or develop*) and (general practice* or GP or family practice* or primary healthcare* or primary care clinic or primary care practice or primary care*) and (qualitative* or interview* or focus group*)) |
| **Embase** | (patient* and (experience* or view* or opinion* or thought* or idea* or perception* or perspective*) and (pharmacy or pharmacist* or pharmac* or clinical pharmacist* or pharmacy service* or clinical pharmacy service* or primary healthcare pharmacist* or primary care pharmac* or practice-based pharmacist* or general practice pharmac* or practice pharmacy professional or non?dispensing pharmacist*) and (implement* or integrat* or develop*) and (general practice* or GP or family practice* or primary healthcare* or primary care clinic or primary care practice or primary care*) and (qualitative* or interview* or focus group*)).mp. [mp=title, abstract, heading word, drug trade name, original title, device manufacturer, drug manufacturer, device trade name, keyword heading word, floating subheading word, candidate term word] |
| **IPA** | (patient* and (experience* or view* or opinion* or thought* or idea* or perception* or perspective*) and (pharmacy or pharmacist* or pharmac* or clinical pharmacist* or pharmacy service* or clinical pharmacy service* or primary healthcare pharmacist* or primary care pharmac* or practice-based pharmacist* or general practice pharmac* or practice pharmacy professional or non?dispensing pharmacist*) and (implement* or integrat* or develop*) and (general practice* or GP or family practice* or primary healthcare* or primary care clinic or primary care practice or primary care*) and (qualitative* or interview* or focus group*)).mp. [mp=title, subject heading word, registry word, abstract, trade name/generic name] |
| **CINAHL** | (patient* and (experience* or view* or opinion* or thought* or idea* or perception* or perspective*) and (pharmacy or pharmacist* or pharmac* or clinical pharmacist* or pharmacy service* or clinical pharmacy service* or primary healthcare pharmacist* or primary care pharmac* or practice-based pharmacist* or general practice pharmac* or practice pharmacy professional or non?dispensing pharmacist*) and (implement* or integrat* or develop*) and (general practice* or GP or family practice* or primary healthcare* or primary care clinic or primary care practice or primary care*) and (qualitative* or interview* or focus group*)) |
| **Scopus** | ALL((patient* and (experience* or view* or opinion* or thought* or idea* or perception* or perspective*) and (pharmacy or pharmacist* or pharmac* or clinical pharmacist* or pharmacy service* or clinical pharmacy service* or primary healthcare pharmacist* or primary care pharmac* or practice-based pharmacist* or general practice pharmac* or practice pharmacy professional or non?dispensing pharmacist*) and (implement* or integrat* or develop*) and (general practice* or GP or family practice* or primary healthcare* or primary care clinic or primary care practice or primary care*) and (qualitative* or interview* or focus group*))) |
| **Web of Science** | (patient* and (experience* or view* or opinion* or thought* or idea* or perception* or perspective*) and (pharmacy or pharmacist* or pharmac* or clinical pharmacist* or pharmacy service* or clinical pharmacy service* or primary healthcare pharmacist* or primary care pharmac* or practice-based pharmacist* or general practice pharmac* or practice pharmacy professional or non?dispensing pharmacist*) and (implement* or integrat* or develop*) and (general practice* or GP or family practice* or primary healthcare* or primary care clinic or primary care practice or primary care*) and (qualitative* or interview* or focus group*)) |
